# Persistence and activity levels of primitive reflexes in young high-level football players

**DOI:** 10.3389/fspor.2024.1409257

**Published:** 2024-07-17

**Authors:** Julie Bastiere, Thibault Lussiana, Damien Young, Cyrille Gindre, Laurent Mourot

**Affiliations:** ^1^ SYNERGIES, Université de Franche-Comté, Besançon, France; ^2^ Plateforme Exercice Performance Santé Innovation, Université de Franche-Comté, Besançon, France; ^3^ ESTAC Association, 5 rue Marie Curie, Troyes, France; ^4^ Research and Development Department, Volodalen, Chavéria, France; ^5^ Research and Development Department, Volodalen SwissSportLab, Aigle, Switzerland; ^6^ Technology University of the Shannon, Midlands Midwest, Thurles Campus, Tipperary, Ireland; ^7^ Department of Biological Sciences, Faculty of Science, Thompson Rivers University, Kamloops, BC, Canada

**Keywords:** primitive reflexes, neuroscience, football, score reflex, neurodevelopment

## Abstract

**Introduction:**

Primitive reflexes (PR) induce involuntary automatic movements in response to specific stimuli. This study aimed to determine the prevalence of active PR in young high-level football players.

**Methodology:**

Sixty-nine national-level football players from a French academy were tested (17.0 ± 1.4 years; 69.6 ± 8.0 kg; 178.9 ± 6.9 cm) to evaluate the persistence of PR, following the methodology of the Institute for Neuro-Physiological Psychology (INPP) and the classification by a global score (GS). Based on the sum of seven tests, each was rated between 0 = null and 4 = max. The GS is classified into five categories from no activity to maximal (0–1 = no activity, 2–7 = low, 8–13 = medium, 14–21 = high, and 22–28 = maximal).

**Result:**

Around two-thirds (68.1%) of players presented active PR at different activity levels. Among them, a small proportion (7.2%) had medium GS, while 60.9% had a low GS. The GS was not dependent on field position or the age of the players (*p *> 0.05). However, playing football in an age category higher than their own was associated with significantly more active primitive reflexes (PR) compared to being in their age category (*p* < 0.01). The results showed that 72.7% of “upgraded” football players had low GS and 18.2% had medium GS, compared to 55.3% and 2.1% in the non-upgraded group.

**Discussion:**

The findings of the current study demonstrate that PR could still be active in a healthy population of high-level football players. Practicing a single sport for years and upgrading players could create a negative environment that can ultimately lead to the activation of otherwise integrated PR.

## 1 Introduction

A football game is characterized by approximately 1,200 acyclical and unpredictable changing actions, including 700 turns, 30–40 jumps or tackles, dribbling, and kicking (1, 2). Football technical skills require cognitive and physical abilities and postural control such as strength and speed (3, 4), resulting in excellent agility and a sense of balance that is a rejection assessment criterion if not effective in the field for talent identification (4). Balance skills require the adjustment of rapid and consistent muscle responses in an ever-changing environment using the synergy of three systems: (a) visual, (b) proprioceptive, and (c) vestibular (5). This trio of postural control tools allows individuals to respond to a specific environment that can be interfered with by active primitive reflexes (PR) (6).

The primitive (or primary) reflexes are automatic (7), stereotyped movement mediated by the brainstem during the first 6 months of the child’s life, which is inhibited during the second 6 months of the child’s life with the maturation of the central nervous system (8). The authors demonstrated the correlation between motor milestones and regression of PR during this period (i.e., rolling and sitting) (9). The PR would be the mechanism used to prepare the body for vertical position and balance by stimulating the vestibular system (10). They will remain within the lower parts of the nervous system to reappear in urgent situations (11). They are historically used by pediatricians for neurological examination during the first year of postnatal life (12). Severe activity of PR after their normal lifespan indicates pathologies such as cerebral palsy (13) or autism spectrum (14). Milder activity of PR is associated with less severe disorders (15) and an indication of a delay in the development of the central nervous system or of immaturity of the vestibular system and associated neural pathways (11, 16). The findings showed that active PR persists in healthy populations, notably in the child population. Pecuch et al. (17) reported that 98% of 44 healthy children (aged 4–6 years) presented at least one active PR. The same research group reported 92.9% of active PR in another cohort of 112 healthy children (aged 4–6 years) in Poland (18). Meanwhile, Hickey and Feldhacker (19) have shown that nearly 100% of 27 preschool children (aged 4–6 years) from the United States had at least one active PR. But also in the older population, research on 27 children with learning difficulties between ages 7.0 and 18.2 years from Poland confirmed that PR could last up to 18 years (20). Furthermore, the same authors reported that the level of activity of active PR could even increase with age. Niklasson et al. (21) showed that PR can be active in the adult population (*n* = 13; 35.21 years ± 10.73) and teenage population (*n* = 100; 12.44 years ± 1.57) in the same proportion.

Studies reported that the active PR can interfere with normal motor development and motor skills (19, 22). Indeed, an active PR can alter neural pathways, decrease motor control (22, 23), and indicate neuromotor immaturity (11). Active PR can also affect learning, and in some cases, this will require more effort and time for good task execution (24, 25).

Each PR has a specific motor response to a stimulus with an involuntary movement or recognized and identifiable behavior (13, 26). The asymmetrical tonic neck reflex (ATNR) appears to be the most delegated PR in performing motor tasks, such as reading difficulty (24, 27), in fine and gross motor control as in writing, creeping, and catching a ball (24). The symmetrical tonic neck reflex (STNR) when actively prolonged in time has negative effects on the visual system such as tracking an object approaching at speed (e.g., catching a ball) (6, 28). The tonic labyrinthine reflex (TLR) and the Moro reflex (MR) have vestibular origins. The MR, when active, can create fear of falling and has a major negative impact on psychological and physiological development (29), while TLR can pose many symptoms described by Goddard Blythe (11) as insecure balance, hypotonus, and spatial problem. These four PR were considered as having a diagnostic value, have already been tested in healthy adult populations, and can be connected to all regulatory systems of balance (vestibular-proprioceptive and visual) that are necessary to perform football at a high level (21, 30, 31). In the field of sports, Bogdanoviča et al. (32) reported that active PR (TLR, STNR, and ATNR) increased the time required to assimilate technical gestures and skills quality in two 7-year-old children that had the same level of swimming ability before lessons. The children with 8% of APR achieved a level of swimming proficiency of 77% compared with 33% for the child with more than 25% of APR. They concluded that to improve swimming ability, tasks should be repeated more frequently and needed more time for children with active PR to achieve comparable results to children of the same age with integrated PR. These three active PR have an incidence on the muscular tonus which will cause a gap between the messages passing from the vestibular system to the body and the body's reaction (retroaction of somatosensory system). There will be a difference between the task performed and the final performance (11).

Thus, it could be hypothesized that during a football match, a player with active PR could show difficulties in reading trajectory or performing the correct gesture and the best tactical choice in a stressful environment. However, to the best of the authors' knowledge, there is no information on the prevalence of active PR in the population of elite football players. Therefore, the main objective of this study was to evaluate whether PR can be still active and their corresponding activity levels in adolescents and adults practicing football.

## 2 Material and methods

### 2.1 Participants

This cross-sectional study included all football players from a French professional club academy who met the inclusion criteria. To be eligible, players must have had over 5 years of training in a sports club prior to joining the academy and must not have had any injuries in the month preceding the evaluation. Players who were injured during the test period or had an injury in the last month were excluded from the study.

A total of 69 male football players participated in this study (age, 17.0 ± 1.5 years; weight, 69.6 ± 8.0 kg; height, 179.0 ± 6.9 cm). These players had been part of the academy for an average of 1.8 ± 1.7 years. The study population was divided based on three criteria: (1) year of birth, to assess age-related integration (Group 1, year of birth), (2) whether a player had been upgraded or not, to evaluate stress-induced reactivation (Group 2, player upgrade status), and (3) playing position on the pitch, to determine activation by different stimuli such as head rotation, head flexion, or extension (Group 3, playing position).

#### 2.1.1 Years of birth

Six groups were established according to the birth year: 6 players born in 2001 (weight, 78.8 ± 7.3 kg; height, 182.7 ± 9.8 cm); 5 players born in 2002 (weight, 66.5 ± 3.5 kg; height, 182.9 ± 5.2 cm); 15 players born in 2003 (weight, 70.6 ± 7.8 kg; height, 179.5 ± 6.4 cm); 19 players born in 2004 (weight, 71.0 ± 7.3 kg; height, 178.3 ± 7.0 cm); 17 players born in 2005 (weight, 65.8 ± 6.2 kg; height, 176.5 ± 7.1 cm), and 16 players born in 2006 (weight, 65.6 ± 5.6 kg; height, 178.2 ± 5.8 cm).

#### 2.1.2 Player upgraded

The players were divided into two groups: 47 players were playing in their age category “non-upgraded” (age, 17.3 ± 1.6 years; weight, 71.9 ± 7.3 kg; height, 179.6 ± 6.9 cm), and 22 players were playing in a higher-age category “upgraded” than their age (age, 16.4 ± 1.1 years; weight, 65.3 ± 5.5 kg; height, 177.6 ± 6.8 cm).

#### 2.1.3 Player position

The player's position was divided into six groups: 10 goalkeepers (age, 17.0 ± 1.4 years; weight, 75.9 ± 7.1 kg; height, 182.8 ± 8.7 cm); 16 central defenders (age, 16.9 ± 1.2 years; weight, 72.0 ± 3.6 kg; height, 182.9 ± 5.2 cm); 8 full backs (age, 16.8 ± 1.6 years; weight, 71.2 ± 3.8 kg; height, 177.5 ± 6.1 cm); 18 midfielders (age, 16.9 ± 1.9 years; weight, 63.9 ± 2.4 kg; height, 175.4 ± 2.7 cm); 12 wingers (age, 17.0 ± 1.8 years; weight, 71.0 ± 1.6 kg; height, 176.1 ± 7.1 cm); and 5 forwards (age, 16.7 ± 1.8 years; weight, 68.0 ± 7.1 kg; height, 180.1 ± 4.7 cm).

### 2.2 Test of primitive reflex

Testing took place during the first week after returning to the academy, following the mid-season break in July. Each player was tested in return to the academy following the same protocol of screening test, including the PR. All football players have been tested by the same operator using the Institute for Neuro-Physiological Psychology (INPP) methodology (6). Four PR described in the introduction were tested with four trials, except for the MR, which was tested once. ATNR was assessed while standing, feet together, arms forward at shoulder height, eyes closed, wrists flexed, and hands relaxed. The test consisted of a rotation of the head on both sides induced by the practitioner (left side, ATNR L; right side, ATNR R). The movement is continuous with a pause for 5–10 s at the end of each rotation or neutral position. Before starting, the practitioner informed the participants that they must keep their arms forward without moving. The involuntary movement is rated as the function of the movement of the hand or arm on the side to which the head was turned. TLR was tested in a standing position, feet together and eyes closed. The test was performed following two manipulations. The first consisted of an extension of the head (TLR EXT) and the second in a flexion (TLR FLX) of the head. The movement is continuous with a pause for 5 s at each end of the movement (i.e., head extension and head flexion). The involuntary movement is rated as the function of loss or alternation of balance.

STNR was tested in a quadruped position (hands below shoulders and knees below hips). The instruction for the participant is to hold the quadruped position during the movement of the head during flexion (SNTR FLX) or extension (STNR EXT). The movement is continuous with a pause for 5 s at each end of the movement (i.e., head extension and head flexion). The involuntary movement is rated as the function of the tremors in one or both arms or hip movements, bending of the arms due to flexion of the head, or arching of the back. MR is tested standing, feet joined, arms forward at shoulder height, eyes closed, wrists flexed, hands relaxed, and the head back. After clearly explaining the test and asking if the participant was ready, the subject was asked to drop into the hands of the practitioner by a back rocker. The involuntary movement is rated as the function of abduction of arms or distress during the test.

The PR were graded on a subjective scale ranging from 0 to 4 (one in one increment) based on the involuntary movement that was observed after a specific task or stimulation. A sample of the scoring system is given in Table 1, and a detailed grading system has been by Sally Goddard Blythe (INPP, Chester, UK) [6]. Level 0 indicates no activity (no involuntary movement) to stimuli during the test, up to 4, which indicates the maximum level of activity of PR (large involuntary movement) to stimuli during the test.

**Table 1:** Sample primitive reflex grading for ATNR.

Primitive reflex	INPP scoring scale
Asymmetrical tonic neck reflex (ANTR) Right and left.(R and L)	0. No movement of the arms in response to head rotation
	1. Slight deviation of the arm(s) up to 12–15° in the direction of head
	2. Rotation 30° rotation of the arms
	3. 45° rotation of the arms
	4. 90° rotation of the arms

To observe and compare the distribution of active PR, we have built on Arnold Capute’s pioneering work on primitive reflex profile and contemporary authors on reflex activity level, which is obtained by adding each score of each test. Based on previous work, the sum of each PR score was performed to have a global score (GS) reflecting the activation percentage of PR: 0–1 = no activity (<5% of activity), 3–7 = low (<25% of activity), 8–13 = medium (<50% of activity), 14–21 = high (<75% of activity), and 22–28 maximal (<100% of activity) of APRs (6, 18, 33).

### 2.3 Data and statistical analyses

All the data exported from the MetaSoft Studio software were merged into a Microsoft Office 365 Excel spreadsheet (Microsoft, Redmond, WA, USA) and computed to be analyzed with a custom RStudio algorithm on the desktop software version 1.4.1106 (RStudio PBC, Boston, MA, USA). No sample size calculation was performed *a priori* due to the lack of pre-existing data in the literature. Descriptive statistics were computed for all variables and are reported as mean ± standard deviation (SD) or as prevalence, expressed as the percentage of football players. The categories were transformed into numerical values (e.g., the player in his category as 0 and upgraded as 1). Pearson's chi-squared tests were used to examine associations between levels of primitive reflex activity and categorical variables including player position, year of birth, and upgrade status. The position game has been divided into six positions (goalkeeper, central Back, fullback, midfielders, winger, and forward). For comparisons involving smaller sample sizes where chi-squared tests were not appropriate (*n* < 5), Fisher's exact tests were employed. Given the small sample sizes in some categories, we calculated the effect size to complement the statistical analysis, whose power may be limited. The effect sizes for these tests were quantified using Cohen's *w*, with effect sizes considered trivial for *w* = 0.10, moderate for *w* = 0.30, and large for *w* = 0.50. The significance threshold was set at *p* < 0.05 for all tests.

## 3 Results

### 3.1 Global score and the score of reflex activities

**Figure 1:**
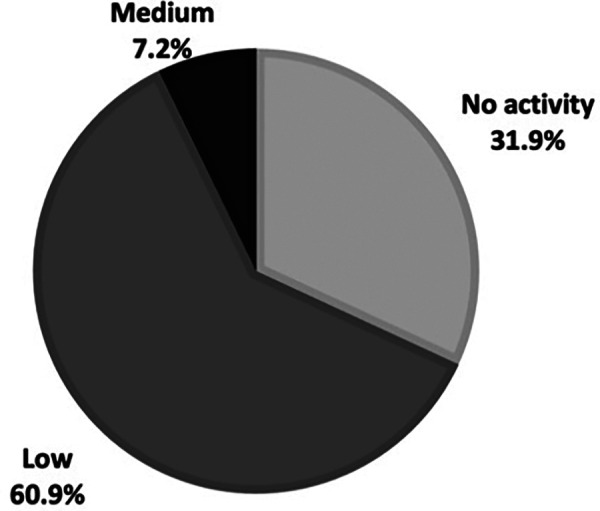
Distribution of the global score of 69 football players of the academy.

The results showed that 42 players had a low GS (60.9%), 5 had a medium GS (7.2%), and 22 had a null GS (31.9%) (Figure 1). No football player had a GS higher than medium (i.e., high and maximal).

Football players with a GS equal to or higher than low had an average of 2 active PR. Moreover, 81.2% of football players demonstrated at least one active PR, and 23.0% had more than 2 active PR.

The distribution of scores was different between the PR tested (*χ*^2^ = 83.579, df = 24, *p* = 1.621 × 10^−8^, Cohen's *w* = 1.87): four of the football players scored at 3 (MR 2.9%; TLR EXT 2.9%; TLR FLX 4.3%; STNR FLX 1.4%), and only two football players (2.9%) obtained the maximum score of 4 in the MR. The TLR EXT, when considering all players who were tested at level 1 or higher, was observed in 55.1% of the football players, making it the most observed persistent PR. Conversely, the STNR EXT was the least observed PR, noted in only 5.8% of the football players (Table 2).

**Table 2:** Distribution of activity levels of primitive reflexes.

PR	*N* % total of integrated primitive reflexes	Score of PR activities			
		*N* % low activity	*N* % medium activity	*N* % high activity	*N* % maximal activity
MR	49 71.0%	10 14.5%	6 8.7%	2 2.9%	2 2.9%
TLR FLX	42 60.9%	15 21.7%	9 13.0%	3 4.3%	0 0.0%
TLR EXT	31 44.9%	20 29.0%	16 23.2%	2 2.9%	0 0.0%
ATNR R	54 75.4%	13 21.7%	2 2.9%	0 0.0%	0 0.0%
ATNR L	49 71.0%	12 18.8%	6 10.1%	0 0.0%	0 0.0%
STNR EXT	65 94.2%	4 5.8%	0 0 0.0%	0 0.0%	0 0.0%
STNR FLX	60 86.9%	8 11.6%	0 0.0%	1 1.4%	0 0.0%

ATNR, asymmetrical tonic neck reflex; STNR, symmetrical tonic neck reflex; TLR, tonic labyrinthine reflex; L, left; R, right; EXT, extension; FLX, flexion.

### 3.2 Global scores: player's position and age of birth

**Figure 2:**
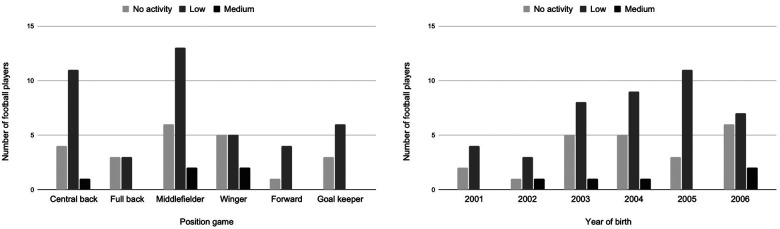
Distribution of the global score of primitive reflexes compared to position game and year of birth.

No significant difference in GS was observed regarding player's playing positions (*χ*^2^ = 3.6146, df = 10, *p* = 0.9, Cohen's *w* = 0.30). Similarly, no significant difference (*χ*^2^ = 11.198, df = 10, *p* = 0.4, Cohen's *w* = 0.40) was observed as regards the age of the players (Figure 2).

### 3.3 Global scores and upgrade

Table 3 shows the distribution of GS within the two groups of upgraded and non-upgraded football players. A significant difference in the distribution of reflex scores was observed between upgraded and non-upgraded players using Fisher's exact test (*p* = 0.006355, Cohen's *w* = 0.40). The proportion of players with a null GS was significantly higher in the non-upgraded (*n* = 20) than that in the upgraded players (*n* = 2) (*p* = 0,005). Conversely, the proportions of players with a low GS did not show any significant differences between the upgraded and non-upgraded players (*p* = 0.2). Among the players with a medium GS tested, the proportion of non-upgraded players was significantly lower (*n* = 1) than that in the upgraded players (*n* = 4) (*p* = 0.03).

**Table 3:** Distribution of global score compared to the upgraded football players.

GS	Non-upgraded players (*n*)	Upgraded players (*n*)
No activity	42.6% (20)	9.1%* (2)
Low	55.3% (26)	72.7% (16)
Medium	2.1% (1)	18.2%* (4)
High	0.0% (0)	0.0% (0)
Maximal	0.0% (0)	0.0% (0)

* Indicates a significant difference between upgraded and non-upgraded football players (*p* < 0.02).

## 4 Discussion

This study aimed to evaluate the persistence and the prevalence of active PR in a population of young high-level football players (age, 17.0 ± 1.5 years). According to previous research on the persistence of PR in healthy children, our results showed that a large proportion of football players involved in this study still have active PR. The presence of PR is even more visible in the upgraded population, who showed more active PR than players who were not upgraded. However, the playing position and calendar age groups did not significantly influence the distribution of active PR. 81.2% of our football players demonstrate at least one PR active while no GS scored higher than medium. These results are consistent with those of a previous study, which was conducted in a healthy preschool and school population that reported no maximal GS (23). However, two midfielder players who were upgraded in the 15- and 17-year categories were found to have the MR at a maximal level. Previous authors also reported that the MR was the most frequently expressed PR at the maximum level (17).

The GS depends on both the level of active PR and the number of active PR. Our results revealed that levels of activity were significantly different according to the PR tested. The STNR EXT was active in 5.7% of football players, while the TLR EXT was active in 54.3%. We observed five active PR rated 3 or more during testing. Previous studies on PR have reported that even a low GS can negatively affect the motor organization in the child population (22). The active PR negatively diminishes the ability to perform physical tasks and learning in young children. Goddard Blythe and Beuret (11) showed that symmetric tonic neck reflex (STNR) affects children when learning the breaststroke: “Each time the head is raised to keep it out of the water, the feet start to sink, making it very difficult to keep the body on the top of water.” Pecuch et al. (17, 18) recently confirmed that active tonic PR is associated with poor balance (reverse and forward) and difficulty jumping over a cord, performing jumping jacks, or rolling around the long axis of the body. Recently, Kalemba et al. (34) showed the correlation between active PR (ATNR, STNR) and clock reading difficulty.

Bogdanoviča et al. (32) reported in a case study on swimming proficiency that only 25% of active PR increase the difficulty of swimming proficiency and learning to swim with the child in otherwise good health and without a learning difficulty. Each of the four tested PR has a distinct effect on motor and learning abilities in the healthy population (35, 36). Indeed, it has been reported that the ATNR can alter the way the pelvis moves and positions itself during walking and is associated with difficulty crossing the midline of the body and asymmetry in the muscle tone (6, 22). The STNR has been linked with difficulties in learning abilities and postural balance (18, 37). The TLR in extension, can alternate not only coordination and posture but also a hypertone due to hyperextension of the posterior chain. Conversely, the TLR in flexion induced a hypotony and a precarious balance (11). The MR is linked to context and environment. Such an activated PR can create feelings of insecurity and danger translated by the creation of uncontrolled movements related to their level of activity (38, 39). The MR may be involved in some skill difficulties, such as, when heading the ball in football. In response to the observed general alignment in the literature regarding primitive reflexes and healthy subjects, it is crucial to consider the specific demographic characteristics of the populations involved. While our findings corroborate the general patterns observed, the demographic nuances of our study's population highlight the need for targeted interventions tailored to specific age groups and activity levels.

An important observation in this study was the distribution of GS in upgraded players who presented a significantly higher rate of active PR than non-upgraded players (Figure 2). While the difference was statistically significant, the effect size was moderate (*w* = 0.40). The specificity of this upgraded population lies in their being the younger players within their team, where the understanding of the game and the technical and physical levels surpass those typical of their age category. This gap between categories could create an emotional factor inappropriate for good motor development or for keeping PR integrated. As noted earlier, the MR has been tested at the maximal level only in upgraded players. MR induces an involuntary response during a rear imbalance, and it could be inconvenient during aerial play, where contact with the opponent could throw the player backward. Moreover, this active PR can induce an environment not only by the creation of stress and fear but also by being followed up by higher levels of cortisol (40). As previously demonstrated, an explanation for higher prevalence could be linked to the fact that the prevalence of active PR decreases with increasing age (41). However, the current results did not point to a specific effect of age (Figure 2). Hence, an alternative explanation could be that the early integration of young football players into higher-age categories could create an inappropriate environment for their physical and emotional development (38, 39). The present results suggested that the playing position in football did not show any significant differences between the GS.

The present findings should be considered with some limitations. The study only focused on male football players aged between 15 and 19 years old. It would be interesting if further research investigated GS in adult women’s football populations. In addition, the methods used to determine GS and to test PR are subjective tests. Although they were conducted in accordance with the recommendations of the INPP screening test (6), there is no evidence of their validity for young adults. Hence, validating them in a future study of this population is necessary. Moreover, the persistence of PR was tested by assessing observable responses to specific stimuli, but we did not consider the specific evaluation of the contribution of the different somatic sub-system, specifically the vestibular system. This should be assessed in future studies.

Future studies should evaluate the relationship between active PR with motor skills in the healthy population. For example, research conducted on PR and motor abilities in children (4–6 years) has shown that the level of activity of PR affects motor skills even if this level is low (11, 23). Indeed, the involuntary movement generated by an active PR might interfere with optimal movement and motor skills (18, 28). However, the current results showed that it was the upgraded football players who had the most active PR. Therefore, such involuntary movement caused by active PR could indicate a specific lack of motor skills or development as well as neuromotor immaturity and is not correlated with the player level. More specifically, the effects of active PR may depend on the situation the player is in. Active PR may have negative effects in one context (e.g., increased imbalance per rotation with the ATNR) and positive effects in another (e.g., enhanced rotation speed with the ATNR).

As a starting point, this study opens a new approach to motor proficiency in high-level football players or sports areas. Indeed, we can hypothesize that the environment induced by high-level demands can create a favorable ground for PR activation or observing an active PR can be an indirect information on possible vestibular system dysfunction. Additionally, it raises the question of whether the football players had initially correctly integrated the PR, but that the high-level practice of a single activity (here football) for years does not allow their integration by the central nervous system to be maintained in the long-term.

In conclusion, the present study showed that PR are still largely active in high-level football players, notably on upgraded players, independently of age and playing position. Further studies are needed to evaluate how and to what extent active PR could be linked to injuries and alter the player’s motor organization and comportment during game and training.

## Data Availability

The original contributions presented in the study are included in the article/Supplementary Material; further inquiries can be directed to the corresponding author.
